# COVID-19 infection triggered idiopathic capillary leak syndrome treated with ECMELLA

**DOI:** 10.1016/j.heliyon.2024.e34693

**Published:** 2024-07-16

**Authors:** Michael Wester, Thomas Drasch, Roland Schneckenpointner, Maik Foltan, Alois Philipp, Thomas Müller, Bernhard Banas, Lars S. Maier, P.D. Matthias Lubnow

**Affiliations:** a University Heart Centre Regensburg, Department of Internal Medicine II, University Hospital of Regensburg, Regensburg, Germany; b Department of Nephrology, University Hospital of Regensburg, Regensburg, Germany; c Department of Cardiothoracic Surgery, University Hospital of Regensburg, Regensburg, Germany

**Keywords:** Case report, VA-ECMO, Impella, COVID19, ISCLS

## Abstract

**Background:**

Idiopathic systemic capillary leak syndrome (ISCLS) is characterized by recurrent systemic capillary leakage and hypovolemic shock.

**Case presentation:**

We report a 59-year-old Caucasian man with ISCLS and persistent hypovolemic and cardiogenic shock after COVID-19 infection. Mechanical circulatory support was provided with veno-arterial extracorporeal membrane oxygenation and a microaxial pump. Massive fluid resuscitation was needed. Subsequent complications prolonged the intensive care treatment. Mechanical circulatory support was needed for 22 days. Cardiac function eventually fully recovered, and the patient survived without neurologic compromise.

**Conclusions:**

This case of severe ISCLS triggered by COVID-19 highlights that even the most severe hypovolemic and cardiogenic shock may be reversible in ISCLS.

## Highlights

• COVID-19 vaccination and infection are potent triggers of ISCLS.
• The lack of a causal therapy warrants maximum supportive therapy including temporary mechanical circulatory support.
• Patients with severe ISCLS are prone to life threatening complications.
• However, ISCLS is potentially completely reversible and good neurological outcome can be achieved.

**Table:** Abbreviations

CK	creatinine kinase
CPC	cerebral performance category
ECG	electrocardiogram
HME filter	heat-moisture-exchange filter
ISCLS	idiopathic capillary leak syndrome
ICU	intensive care unit
IVIG	intravenous immunoglobulin
VA-ECMO	veno-arterial extracorporeal membrane oxygenation

## 1 Case

### 1.1 Background

Idiopathic systemic capillary leak syndrome (ISCLS) is a rare disease that is characterized by recurrent episodes of capillary leakage leading to hemoconcentration, hypoalbuminemia, generalized edema, and hypotension or shock. Those episodes can be triggered by infections or intense physical exertion. The episodes vary in severity and length, are usually self-limiting but can be lethal. The underlying mechanisms are insufficiently understood and no specific treatment exists [1].

To the best of our knowledge, this case is the most severe case of ISCLS that a patient has survived (for reviews of published case series see Refs. [[2], [3], [4]]). It highlights that (1) COVID-19 infection is a potent trigger of ISCLS episodes and that (2) even the most severe ICLS episodes including prolonged cardiogenic shock can be fully reversible. Thus maximal supportive therapy including extracorporal mechanical life support are warranted.

## 2 Case report

**Fig. 1:**
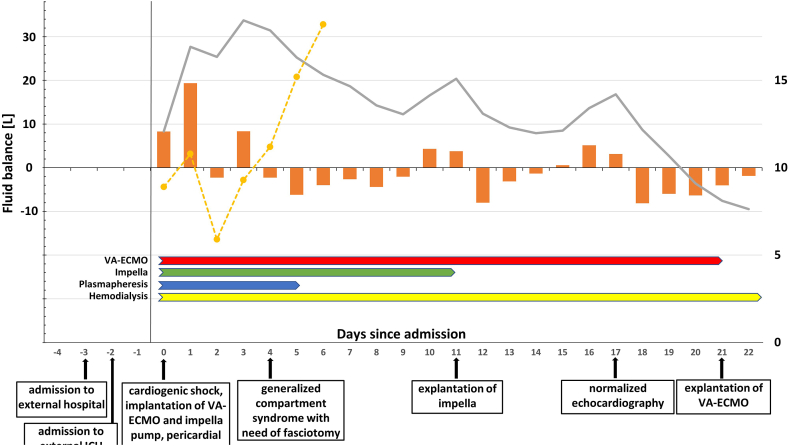
Schematic overview of the intensive care stay. Orange bars indicate daily fluid balance and the grey line indicates total fluid balance (left y-axis). The dotted yellow line indicates daily mean blood pressure amplitude (right y-axis). The arrows below the graph indicate the duration of VA-EMCO (red), Impella (green), plasmapheresis (blue), and hemodialysis (yellow). The boxes below the graph indicate important clinical events. (For interpretation of the references to colour in this figure legend, the reader is referred to the Web version of this article.)

A 59-year-old Caucasian man with COVID-19 infection (PCR testing from bronchoalveolar lavage fluid: Omikron BA.2.9/21L) and persistent hypovolemic and cardiogenic shock was transferred to our ECMO Centre from an external hospital after resuscitation and intubation. COCID-19 symptoms such as mild coughing, fatigue, and a sore throat had occurred 1–2 days before presentation to the externa hospital. The reason for the admission to the external hospital had, however, not been COVID-19-related symptoms but a collapse. At the time of transfer to our centre, echocardiography showed global akinesia without valvular lesions. Pericardial effusion was treated with drainage of 500 mL serous fluid. Coronary angiography ruled out coronary heart disease. ECG showed sinus rhythm. Venoarterial extracorporeal membrane oxygenation (VA-ECMO) was implanted. Despite high doses of epinephrin and levosimendan, the patient had no cardiac output with the invasive blood pressure amplitude intermittently being <3 mmHg for hours within the first three days. The mean arterial blood pressure during the first 5 days of treatment was 65–70 mmHg (the blood pressure amplitude is shown in the Fig. 1). Ventricular venting was established with a microaxial pump (Impella CP® with SmartAssist® Abiomed). After day 4, blood pressure amplitude increased slowly, however, the patient still required catecholamines.

Myocardial biopsy showed slight interstitial edema with fibrin deposits in blood vessels without histological myocarditis. To account for a wide range of possible differential diagnoses, we performed PCR-testing for cardiotrope viruses. The biopsy was positive for HHV-6. Computed tomography showed bilateral pleural effusions, Covid-19 typical ground-glass opacifications, and ascites; no pulmonary artery embolism. Hemoconcentration (hemoglobin 21 g/dl) and hypoalbuminemia (16 g/L) were present.

Initially aggressive fluid resuscitation with crystalloid solution was needed to maintain sufficient mean arterial pressure. The patient gained 34 kg due to positive balance in the first four days. This led to generalized edema and rhabdomyolysis with rising creatinine kinase (CK) due to compartment syndrome. Acute renal failure requiring hemodialysis occurred due to shock and rhabdomyolysis. As CK levels rose above 100 000 U/L (which is the upper limit that is reported by our clinical laboratory) and bladder pressure increased to a maximum of 26 mmHg, indicating progressive generalized compartment syndrome, fasciotomy of the upper and lower limbs as well as decompressive laparotomy were performed. After three days, CK fell to levels <100 000 U/L and then slowly normalized over the next weeks (day 8: 89 459 U/L; day 10: 33 457 U/L; day 16: 1 300 U/L; normalized values on day 32). In accordance, lactate dehydrogenase peaked on day 5 (3 673 U/L), started to slowly fall on day 8 (1 644 U/L), and completely normalized on day 86. Cardiac output recovered and the Impella device was explanted on day 12. At day 18 of ECMO therapy, the patient had regained a normal left ventricular ejection fraction and normal right ventricular function so that VA-ECMO was explanted on day 22.

Plasmapheresis was performed for six days (days 1–5). High dose steroid therapy was administered and slowly tapered (prednisolone 500 mg on day 1, then 100 mg/day for day 2–7; hydrocortisone 10 mg/h from day 1, then slowly tapered until day 26). Monthly intravenous immunoglobulin infusion was started (initially 30 g/d on days 5–8, then 30 g monthly). Despite the mechanical resuscitation and the prolonged intensive care stay, neurologic outcome was good (CPC 1). The patient could be mobilized to a seating position and was transferred for neurologic rehabilitation (day 124). The Fig. 1 shows the clinical course and treatment of the patient.

On day 8, the patient developed severe bilateral broncho-pulmonary aspergillosis which did not sufficiently respond to the prophylactic systemic antifungal therapy with caspofungin. Intravenous posaconazole was added and because of severe bronchial involvement nebulized voriconazole therapy was administered. The mycosis was eventually regressive and gas exchange improved so that ventilatory weaning was started and the patient was extubated on day 68.

The clinical course over the first days and the patient's characteristic medical history with recurrent exacerbations due to viral infections matched previous case reports of ISCLS [1]. Since the age of 56, the patient had had episodes of self-limiting hypotension, peripheral edema, and weight gain after minor infections or vaccinations, sometimes requiring hospitalization. In combination with case reports describing COVID-19 as a possible trigger of ISCLS [2], this prompted the diagnosis of Covid-19 triggered systemic idiopathic capillary leak syndrome. The diagnosis of ISCLS had not been made previously.

ISCLS is a clinical diagnosis lacking specific biomarkers. Secondary causes of capillary leak syndrome and differential diagnoses were considered, but diagnostics gave no indication for sepsis, autoimmune diseases, hemophagocytic lymphohistiocytosis, systemic mastocytosis, or hereditary angioedema. Like in other ISCLS cases, our patient showed monoclonal gammopathy (type IgG lambda), although the relevance of this is still unclear [1]. HHV-6 genome integration could be detected. To account for all potential causes of heart failure, we chose to treat a possible HHV-6 reactivation, we administered systemic ganciclovir (day 8–69).

## 3 Discussion

The episode of ISCLS was triggered by a COVID-19 infection. The patient had previously received a mRNA-based vaccination dose. However, because of a lighter episode of ISCLS, the second dose was not administered. Our report adds to the finding that SARS-CoV2 [2] and SARS-CoV2 vaccination [5] can be potent triggers for acute episodes of ISCLS. Although data is still limited, it seems that the mortality is especially high in COVID-19 triggered episodes reaching up to 50 % [2,6]. Therefore, special emphasis should be on preventive measures in patients with known ISCLS such as general prevention of COVID19 infection and maintaining constant IVIG prophylaxis [2,3].

As there is no causal treatment available for acute ISCLS [1,3] and the episodes are usually self-limiting within several days, the treatment strategy mainly consists of fluid resuscitation during the leaky phase and then of controlling the complications such as edema, compartment syndrome, rhabdomyolysis, renal insufficiency, and cardiac failure [4,[7], [8], [9], [10]]. As the exact pathology is insufficiently understood, targeted therapy is not possible. Different treatment strategies including inotropic agents, methylene blue, anti-inflammatory agents (including corticosteroids, anti-VEGF antibodies, anti-TNFα antibodies), theophyllin, or high dose IVIG have been employed with varying efficacy [1]. However, data regarding effectiveness depend on small case series and good evidence is lacking. In combination with the self-limiting nature of ISCLS episodes, this highlights the importance of maximal supportive therapy until restoration of ISCLS episodes begins.

Cardiac edema and cardiac failure can exacerbate the clinical situation. VA-ECMO in combination with Impella pump has been utilized to bridge persisting shock [5,11]. In those cases, cardiac function recovered quickly within one week. Recovery in our patient was more prolonged and took 17 days. Endomyocardial biopsy showed HHV-6 genome integration. Viral genomes have been implicated in the genesis of reduced left ventricular ejection fraction [12]. It is unknown if and how HHV-6 genome integration should be treated, especially in the context of ISCLS. However, as there was no histological evidence of myocarditis, we assume that the cardiogenic shock was primarily caused by ISCLS and cardiac edema. Even though the likelihood of HHV-6 reactivation being the trigger for heart failure in this patient was small, we chose to treat HHV-6 as the clinical situation was dire and we decided to treat every possible trigger.

On day 8 the patient developed an extensive pulmonary aspergillosis which did not respond sufficiently to intravenous antifungal therapy. We therefore administered nebulized voriconazole [13]. Frequent bronchoscopy revealed a decrease in fungal pneumonia and on day 83 Aspergillus antigen was no longer detectable in the patient's serum or bronchial fluid. As there is no specific formula for nebulization available, the intravenous formula was nebulized. Nebulized voriconazole caused repetitive obstructions of the HME-filter in the expiratory limb before the ventilator. Therefore, nursing staff was advised to routinely change filters after each nebulization to avoid this complication.

## 4 Conclusion

Our case shows that (1) COVID-19 vaccination and infection are potent triggers of ISCLS. (2) The lack of a causal therapy warrants maximum supportive therapy including temporary mechanical circulatory support. (3) Patients with severe ISCLS are prone to life threatening complications. However, (4) ISCLS is potentially completely reversible and good neurological outcome can be achieved.

## Ethics approval

Not applicable.

## Consent for publication

Written informed consent was obtained from the patient for publication of this case report and any accompanying images. A copy of the written consent is available for review by the Editor-in-Chief of this journal.

## Availability of data and materials

No data associated with this case report has been deposited into a publicly available repository. All data relating to this case report has been included in the article and the figure.

## Funding

The authors received no funding for this work.

## CRediT authorship contribution statement

**Michael Wester:** Writing – original draft, Project administration, Methodology, Investigation, Formal analysis, Data curation, Conceptualization. **Thomas Drasch:** Writing – original draft, Methodology, Investigation, Formal analysis, Data curation, Conceptualization. **Roland Schneckenpointner:** Writing – review & editing, Investigation, Data curation. **Maik Foltan:** Writing – review & editing, Investigation, Data curation. **Alois Philipp:** Writing – review & editing, Investigation, Data curation. **Thomas Muller:** Writing – review & editing, Investigation, Data curation. **Bernhard Banas:** Writing – review & editing, Resources. **Lars S. Maier:** Writing – review & editing, Resources. **P.D. Matthias Lubnow:** Writing – review & editing, Supervision, Conceptualization.

## Declaration of competing interest

The authors declare that they have no known competing financial interests or personal relationships that could have appeared to influence the work reported in this paper.

## References

[1] Druey K.M., Parikh S.M. (2017). Idiopathic systemic capillary leak syndrome (Clarkson disease). J. Allergy Clin. Immunol..

[2] Buj M., Morales-Varas G., Pedrosa-Guerrero A., Alonso-Ciria E. (2022). Systemic capillary leak syndrome after SARS-CoV-2 infection and after COVID-19 vaccination: a scoping review in relation to a clinical case. Rev. Clin. Esp..

[3] Pineton de Chambrun M., Luyt C.-E., Beloncle F., Gousseff M., Mauhin W., Argaud L., Ledochowski S., Moreau A.-S., Sonneville R., Verdière B. (2017). The clinical picture of severe systemic capillary-leak syndrome episodes requiring ICU admission. Crit. Care Med..

[4] Gousseff M., Arnaud L., Lambert M., Hot A., Hamidou M., Duhaut P., Papo T., Soubrier M., Ruivard M., Malizia G. (2011). The systemic capillary leak syndrome: a case series of 28 patients from a European registry. Ann. Intern. Med..

[5] Araki T., Morimoto R., Ito R., Mizutani T., Kimura Y., Kazama S., Oishi H., Kuwayama T., Hiraiwa H., Kondo T. (2022). A case of systemic capillary leak syndrome with severe cardiac dysfunction after mRNA vaccination for COVID-19. CJC Open.

[6] Cheung P.C., Eisch A.R., Maleque N., Polly D.M., Auld S.C., Druey K.M. (2021). Fatal exacerbations of systemic capillary leak syndrome complicating coronavirus disease. Emerg. Infect. Dis..

[7] Dolberg-Stolik O.C., Putterman C., Rubinow A., Rivkind A.I., Sprung C.L. (1993). Idiopathic capillary leak syndrome complicated by massive rhabdomyolysis. Chest.

[8] Matsumura M., Kakuchi Y., Hamano R., Kitajima S., Ueda A., Kawano M., Yamagishi M. (2007). Systemic capillary leak syndrome associated with compartment syndrome. Intern. Med..

[9] Ortega F., Carboni Bisso I., Fernandez Ceballos I., Montserrat Rivera A., Tisminetzky M., Dianti J., San Román E., Villarroel S., Di Stefano S., Las Heras M. (2020). Utilización de membrana de circulación extracorpórea en síndrome de leak capilar idiopático: reporte de un caso. Rev. Fac. Cien. Med. Univ. Nac Cordoba.

[10] Wu M.A., Catena E., Cogliati C., Ottolina D., Castelli A., Rech R., Fossali T., Ippolito S., Brucato A.L., Colombo R. (2020). Myocardial edema in paroxysmal permeability disorders: the paradigm of Clarkson's disease. J. Crit. Care.

[11] Arvanitis M., Tuday E., Florido R., Hsu S., Choi C.W., Sharma K., Schulman S.P. (2019). Systemic capillary leak syndrome presenting with fulminant recurrent cardiogenic shock. Circ. Heart Fail..

[12] Kühl U., Pauschinger M., Noutsias M., Seeberg B., Bock T., Lassner D., Poller W., Kandolf R., Schultheiss H.-P. (2005). High prevalence of viral genomes and multiple viral infections in the myocardium of adults with "idiopathic" left ventricular dysfunction. Circulation.

[13] Liao Q., Lam J.K.W. (2021). Inhaled antifungal agents for the treatment and prophylaxis of pulmonary mycoses. Curr. Pharmaceut. Des..

